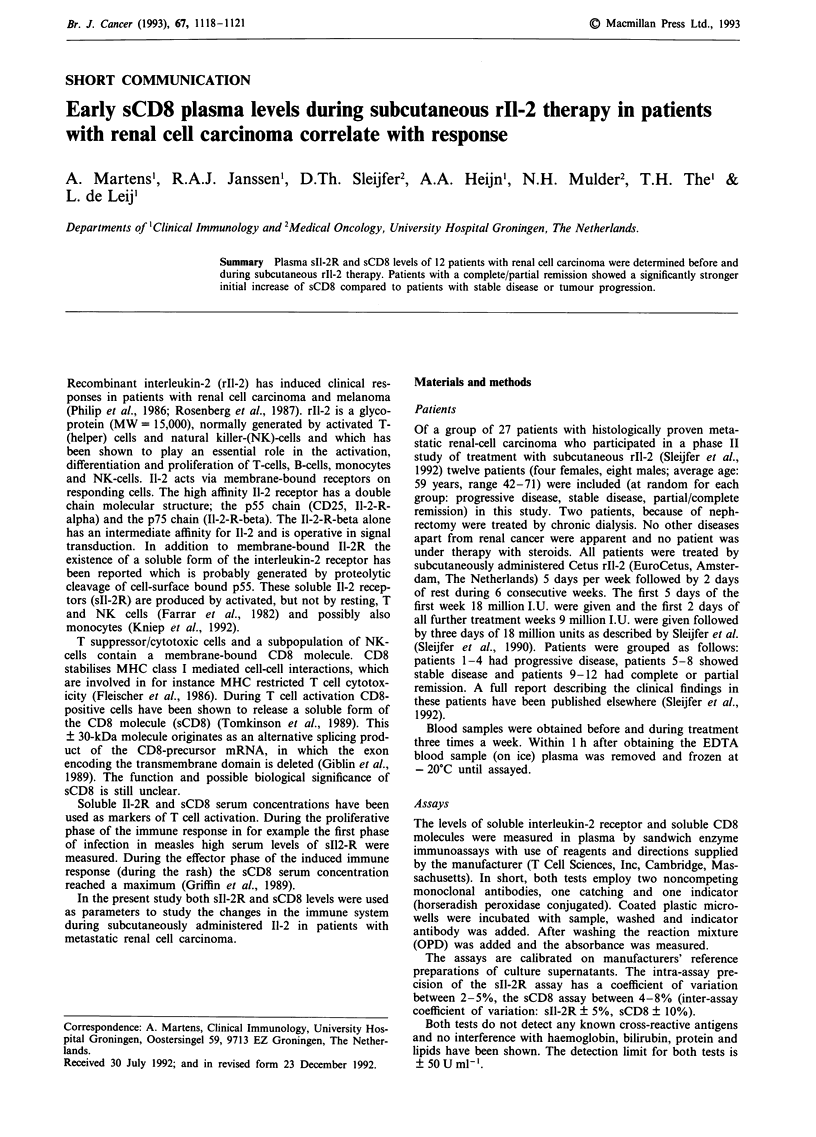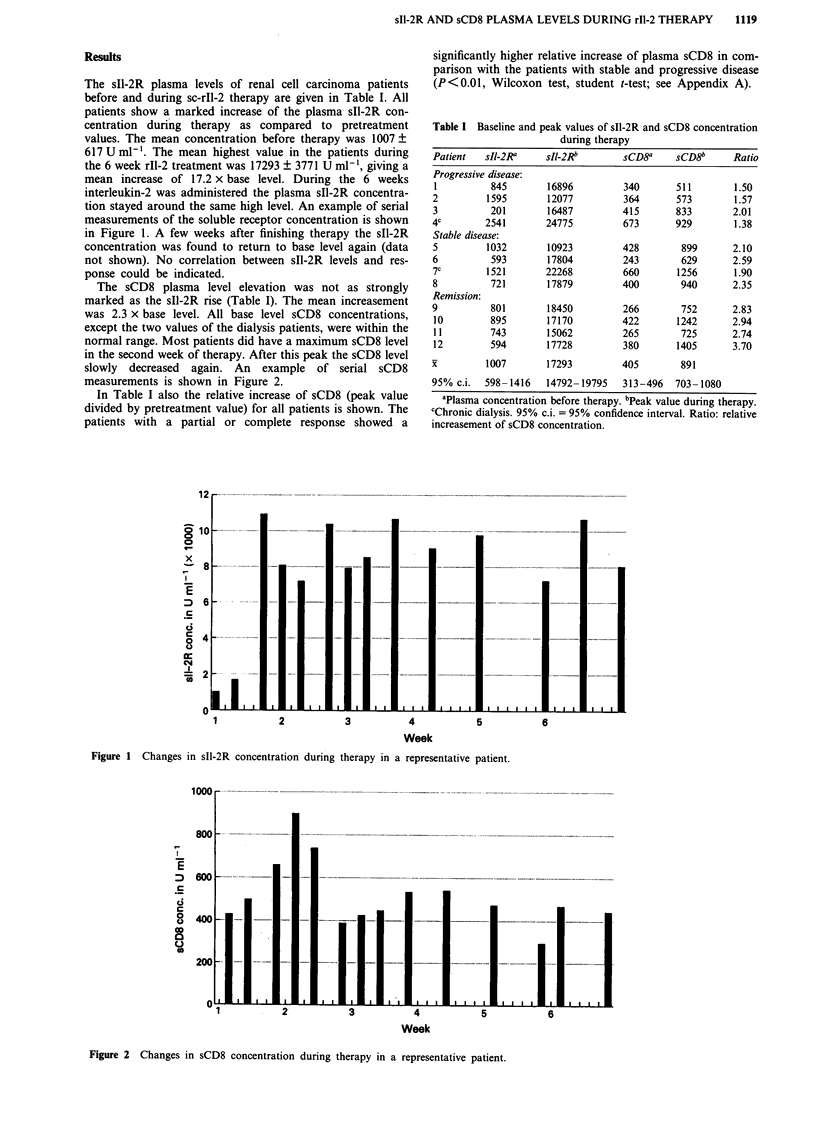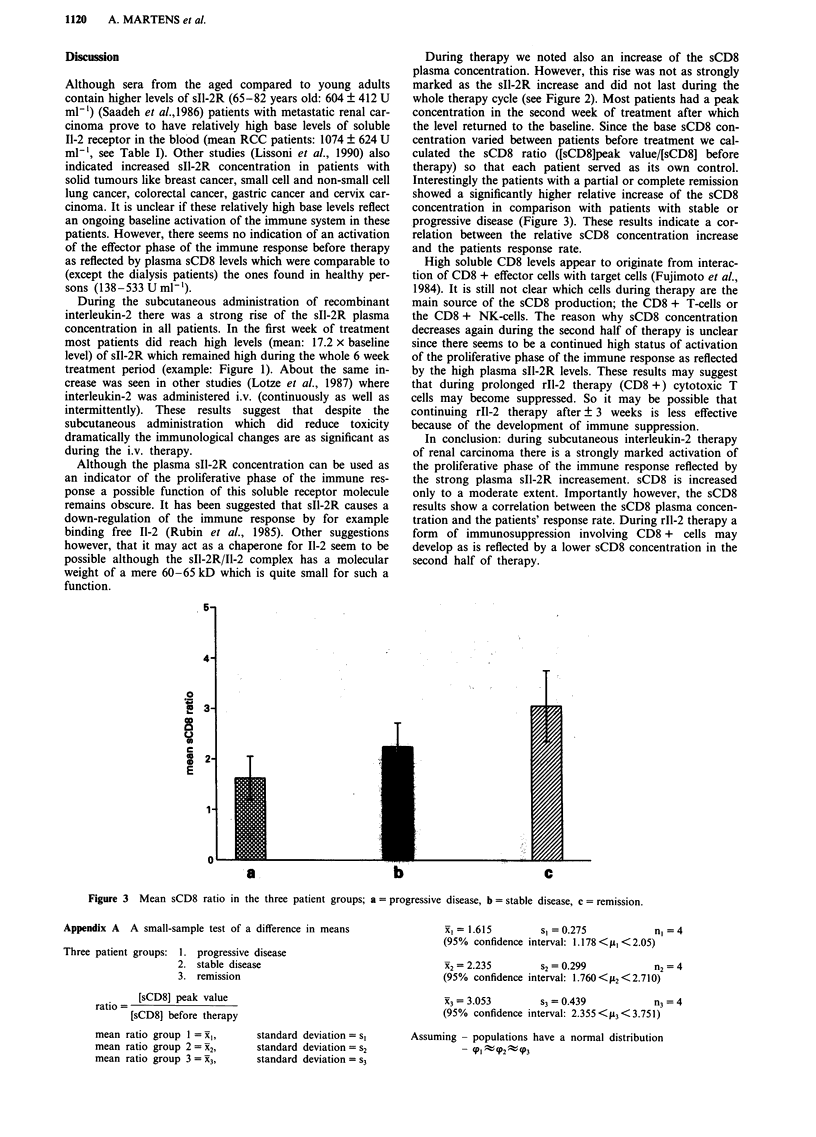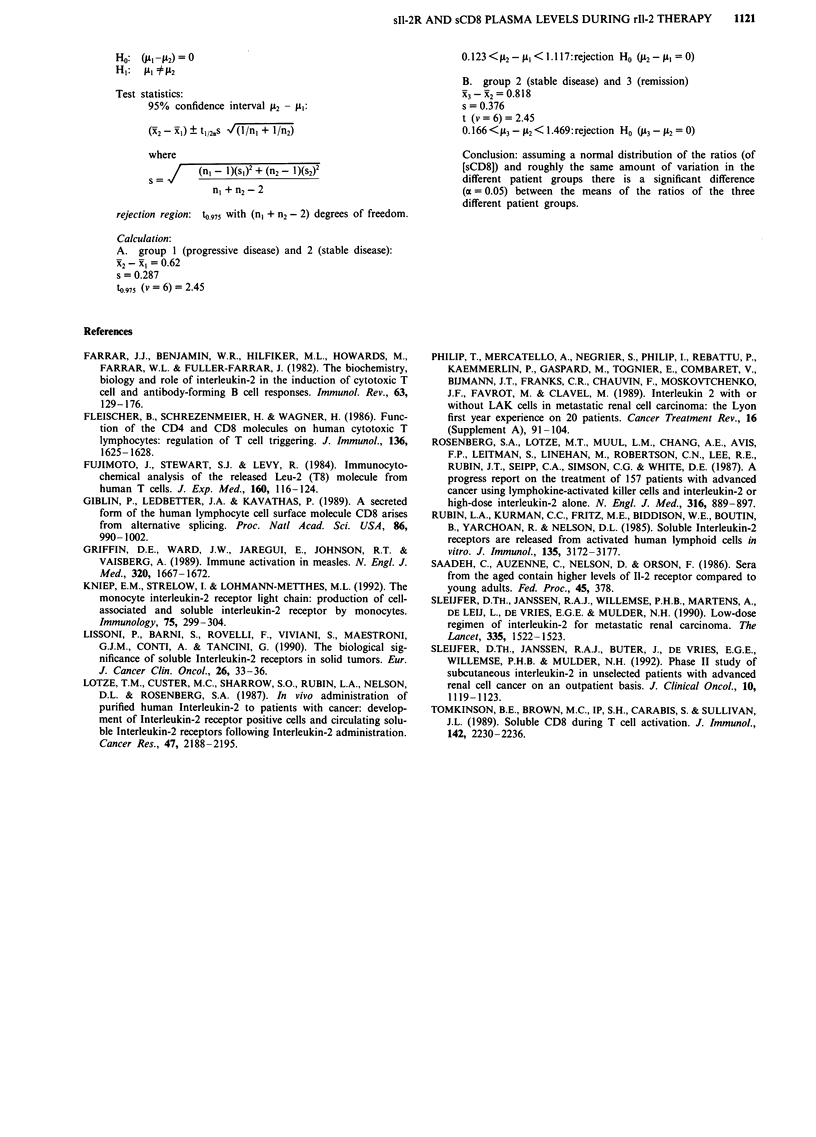# Early sCD8 plasma levels during subcutaneous rIl-2 therapy in patients with renal cell carcinoma correlate with response.

**DOI:** 10.1038/bjc.1993.205

**Published:** 1993-05

**Authors:** A. Martens, R. A. Janssen, D. T. Sleijfer, A. A. Heijn, N. H. Mulder, T. H. The, L. de Leij

**Affiliations:** Department of Clinical Immunology, University Hospital Groningen, The Netherlands.

## Abstract

Plasma sIl-2R and sCD8 levels of 12 patients with renal cell carcinoma were determined before and during subcutaneous rIl-2 therapy. Patients with a complete/partial remission showed a significantly stronger initial increase of sCD8 compared to patients with stable disease or tumour progression.


Br. J. Cancer (1993), 67, 1118–1121 © Macmillan Press Ltd., 1993

SHORT COMMUNICATION

### Early sCD8 plasma levels during subcutaneous rIl-2 therapy in patients with renal cell carcinoma correlate with response

A. Martens[1], R.A.J. Janssen[1], D.Th. Sleijfer[2], A.A. Heijn[1], N.H. Mulder[2], T.H. The[1] & L. de Leij[1]

*Departments of [1]Clinical Immunology and [2]Medical Oncology, University Hospital Groningen, The Netherlands.*

**Summary** Plasma sIl-2R and sCD8 levels of 12 patients with renal cell carcinoma were determined before and during subcutaneous rIl-2 therapy. Patients with a complete/partial remission showed a significantly stronger initial increase of sCD8 compared to patients with stable disease or tumour progression.

Recombinant interleukin-2 (rIl-2) has induced clinical responses in patients with renal cell carcinoma and melanoma (Philip *et al.*, 1986; Rosenberg *et al.*, 1987). rIl-2 is a glycoprotein (MW = 15,000), normally generated by activated T-(helper) cells and natural killer-(NK)-cells and which has been shown to play an essential role in the activation, differentiation and proliferation of T-cells, B-cells, monocytes and NK-cells. Il-2 acts via membrane-bound receptors on responding cells. The high affinity Il-2 receptor has a double chain molecular structure; the p55 chain (CD25, Il-2-R-alpha) and the p75 chain (Il-2-R-beta). The Il-2-R-beta alone has an intermediate affinity for Il-2 and is operative in signal transduction. In addition to membrane-bound Il-2R the existence of a soluble form of the interleukin-2 receptor has been reported which is probably generated by proteolytic cleavage of cell-surface bound p55. These soluble Il-2 receptors (sIl-2R) are produced by activated, but not by resting, T and NK cells (Farrar *et al.*, 1982) and possibly also monocytes (Kniep *et al.*, 1992).

T suppressor/cytotoxic cells and a subpopulation of NK-cells contain a membrane-bound CD8 molecule. CD8 stabilises MHC class I mediated cell-cell interactions, which are involved in for instance MHC restricted T cell cytotoxicity (Fleischer *et al.*, 1986). During T cell activation CD8-positive cells have been shown to release a soluble form of the CD8 molecule (sCD8) (Tomkinson *et al.*, 1989). This ± 30-kDa molecule originates as an alternative splicing product of the CD8-precursor mRNA, in which the exon encoding the transmembrane domain is deleted (Giblin *et al.*, 1989). The function and possible biological significance of sCD8 is still unclear.

Soluble Il-2R and sCD8 serum concentrations have been used as markers of T cell activation. During the proliferative phase of the immune response in for example the first phase of infection in measles high serum levels of sIl2-R were measured. During the effector phase of the induced immune response (during the rash) the sCD8 serum concentration reached a maximum (Griffin *et al.*, 1989).

In the present study both sIl-2R and sCD8 levels were used as parameters to study the changes in the immune system during subcutaneously administered Il-2 in patients with metastatic renal cell carcinoma.

Correspondence: A. Martens, Clinical Immunology, University Hospital Groningen, Oostersingel 59, 9713 EZ Groningen, The Netherlands.
Received 30 July 1992; and in revised form 23 December 1992.

### Materials and methods

### *Patients*

Of a group of 27 patients with histologically proven metastatic renal-cell carcinoma who participated in a phase II study of treatment with subcutaneous rIl-2 (Sleijfer *et al.*, 1992) twelve patients (four females, eight males; average age: 59 years, range 42–71) were included (at random for each group: progressive disease, stable disease, partial/complete remission) in this study. Two patients, because of nephrectomy were treated by chronic dialysis. No other diseases apart from renal cancer were apparent and no patient was under therapy with steroids. All patients were treated by subcutaneously administered Cetus rIl-2 (EuroCetus, Amsterdam, The Netherlands) 5 days per week followed by 2 days of rest during 6 consecutive weeks. The first 5 days of the first week 18 million I.U. were given and the first 2 days of all further treatment weeks 9 million I.U. were given followed by three days of 18 million units as described by Sleijfer *et al.* (Sleijfer *et al.*, 1990). Patients were grouped as follows: patients 1–4 had progressive disease, patients 5–8 showed stable disease and patients 9–12 had complete or partial remission. A full report describing the clinical findings in these patients have been published elsewhere (Sleijfer *et al.*, 1992).

Blood samples were obtained before and during treatment three times a week. Within 1 h after obtaining the EDTA blood sample (on ice) plasma was removed and frozen at − 20°C until assayed.

### *Assays*

The levels of soluble interleukin-2 receptor and soluble CD8 molecules were measured in plasma by sandwich enzyme immunoassays with use of reagents and directions supplied by the manufacturer (T Cell Sciences, Inc, Cambridge, Massachusetts). In short, both tests employ two noncompeting monoclonal antibodies, one catching and one indicator (horseradish peroxidase conjugated). Coated plastic microwells were incubated with sample, washed and indicator antibody was added. After washing the reaction mixture (OPD) was added and the absorbance was measured.

The assays are calibrated on manufacturers' reference preparations of culture supernatants. The intra-assay precision of the sIl-2R assay has a coefficient of variation between 2–5%, the sCD8 assay between 4–8% (inter-assay coefficient of variation: sIl-2R ± 5%, sCD8 ± 10%).

Both tests do not detect any known cross-reactive antigens and no interference with haemoglobin, bilirubin, protein and lipids have been shown. The detection limit for both tests is ± 50 U $ml^{-1}$.

### Results

The sIl-2R plasma levels of renal cell carcinoma patients before and during sc-rIl-2 therapy are given in Table I. All patients show a marked increase of the plasma sIl-2R concentration during therapy as compared to pretreatment values. The mean concentration before therapy was 1007 ± 617 U ml$^{-1}$. The mean highest value in the patients during the 6 week rIl-2 treatment was 17293 ± 3771 U ml$^{-1}$, giving a mean increase of 17.2 × base level. During the 6 weeks interleukin-2 was administered the plasma sIl-2R concentration stayed around the same high level. An example of serial measurements of the soluble receptor concentration is shown in Figure 1. A few weeks after finishing therapy the sIl-2R concentration was found to return to base level again (data not shown). No correlation between sIl-2R levels and response could be indicated.

The sCD8 plasma level elevation was not as strongly marked as the sIl-2R rise (Table I). The mean increasement was 2.3 × base level. All base level sCD8 concentrations, except the two values of the dialysis patients, were within the normal range. Most patients did have a maximum sCD8 level in the second week of therapy. After this peak the sCD8 level slowly decreased again. An example of serial sCD8 measurements is shown in Figure 2.

In Table I also the relative increase of sCD8 (peak value divided by pretreatment value) for all patients is shown. The patients with a partial or complete response showed a significantly higher relative increase of plasma sCD8 in comparison with the patients with stable and progressive disease ($P<0.01$, Wilcoxon test, student $t$-test; see Appendix A).

**Table I** Baseline and peak values of sIl-2R and sCD8 concentration during therapy

| *Patient* | *sIl-2R*[a] | *sIl-2R*[b] | *sCD8*[a] | *sCD8*[b] | *Ratio* |
|---|---|---|---|---|---|
| *Progressive disease*: | | | | | |
| 1 | 845 | 16896 | 340 | 511 | 1.50 |
| 2 | 1595 | 12077 | 364 | 573 | 1.57 |
| 3 | 201 | 16487 | 415 | 833 | 2.01 |
| 4[c] | 2541 | 24775 | 673 | 929 | 1.38 |
| *Stable disease*: | | | | | |
| 5 | 1032 | 10923 | 428 | 899 | 2.10 |
| 6 | 593 | 17804 | 243 | 629 | 2.59 |
| 7[c] | 1521 | 22268 | 660 | 1256 | 1.90 |
| 8 | 721 | 17879 | 400 | 940 | 2.35 |
| *Remission*: | | | | | |
| 9 | 801 | 18450 | 266 | 752 | 2.83 |
| 10 | 895 | 17170 | 422 | 1242 | 2.94 |
| 11 | 743 | 15062 | 265 | 725 | 2.74 |
| 12 | 594 | 17728 | 380 | 1405 | 3.70 |
| $\bar{x}$ | 1007 | 17293 | 405 | 891 | |
| 95% c.i. | 598–1416 | 14792–19795 | 313–496 | 703–1080 | |

[a]Plasma concentration before therapy. [b]Peak value during therapy. [c]Chronic dialysis. 95% c.i. = 95% confidence interval. Ratio: relative increasement of sCD8 concentration.

**Figure 1** Changes in sIl-2R concentration during therapy in a representative patient.

**Figure 2** Changes in sCD8 concentration during therapy in a representative patient.

### Discussion

Although sera from the aged compared to young adults contain higher levels of sIl-2R (65–82 years old: 604 ± 412 U $ml^{-1}$) (Saadeh *et al.*,1986) patients with metastatic renal carcinoma prove to have relatively high base levels of soluble Il-2 receptor in the blood (mean RCC patients: 1074 ± 624 U $ml^{-1}$, see Table I). Other studies (Lissoni *et al.*, 1990) also indicated increased sIl-2R concentration in patients with solid tumours like breast cancer, small cell and non-small cell lung cancer, colorectal cancer, gastric cancer and cervix carcinoma. It is unclear if these relatively high base levels reflect an ongoing baseline activation of the immune system in these patients. However, there seems no indication of an activation of the effector phase of the immune response before therapy as reflected by plasma sCD8 levels which were comparable to (except the dialysis patients) the ones found in healthy persons (138–533 U $ml^{-1}$).

During the subcutaneous administration of recombinant interleukin-2 there was a strong rise of the sIl-2R plasma concentration in all patients. In the first week of treatment most patients did reach high levels (mean: 17.2 × baseline level) of sIl-2R which remained high during the whole 6 week treatment period (example: Figure 1). About the same increase was seen in other studies (Lotze *et al.*, 1987) where interleukin-2 was administered i.v. (continuously as well as intermittently). These results suggest that despite the subcutaneous administration which did reduce toxicity dramatically the immunological changes are as significant as during the i.v. therapy.

Although the plasma sIl-2R concentration can be used as an indicator of the proliferative phase of the immune response a possible function of this soluble receptor molecule remains obscure. It has been suggested that sIl-2R causes a down-regulation of the immune response by for example binding free Il-2 (Rubin *et al.*, 1985). Other suggestions however, that it may act as a chaperone for Il-2 seem to be possible although the sIl-2R/Il-2 complex has a molecular weight of a mere 60–65 kD which is quite small for such a function.

During therapy we noted also an increase of the sCD8 plasma concentration. However, this rise was not as strongly marked as the sIl-2R increase and did not last during the whole therapy cycle (see Figure 2). Most patients had a peak concentration in the second week of treatment after which the level returned to the baseline. Since the base sCD8 concentration varied between patients before treatment we calculated the sCD8 ratio ([sCD8]peak value/[sCD8] before therapy) so that each patient served as its own control. Interestingly the patients with a partial or complete remission showed a significantly higher relative increase of the sCD8 concentration in comparison with patients with stable or progressive disease (Figure 3). These results indicate a correlation between the relative sCD8 concentration increase and the patients response rate.

High soluble CD8 levels appear to originate from interaction of CD8 + effector cells with target cells (Fujimoto *et al.*, 1984). It is still not clear which cells during therapy are the main source of the sCD8 production; the CD8 + T-cells or the CD8 + NK-cells. The reason why sCD8 concentration decreases again during the second half of therapy is unclear since there seems to be a continued high status of activation of the proliferative phase of the immune response as reflected by the high plasma sIl-2R levels. These results may suggest that during prolonged rIl-2 therapy (CD8 +) cytotoxic T cells may become suppressed. So it may be possible that continuing rIl-2 therapy after ± 3 weeks is less effective because of the development of immune suppression.

In conclusion: during subcutaneous interleukin-2 therapy of renal carcinoma there is a strongly marked activation of the proliferative phase of the immune response reflected by the strong plasma sIl-2R increasement. sCD8 is increased only to a moderate extent. Importantly however, the sCD8 results show a correlation between the sCD8 plasma concentration and the patients' response rate. During rIl-2 therapy a form of immunosuppression involving CD8 + cells may develop as is reflected by a lower sCD8 concentration in the second half of therapy.

**Figure 3** Mean sCD8 ratio in the three patient groups; **a** = progressive disease, **b** = stable disease, **c** = remission.

**Appendix A** A small-sample test of a difference in means

Three patient groups: 1. progressive disease

2. stable disease

3. remission

$$\text{ratio} = \frac{[\text{sCD8}]\ \text{peak value}}{[\text{sCD8}]\ \text{before therapy}}$$

mean ratio group 1 = $\bar{x}_1$, standard deviation = $s_1$

mean ratio group 2 = $\bar{x}_2$, standard deviation = $s_2$

mean ratio group 3 = $\bar{x}_3$, standard deviation = $s_3$

$\bar{x}_1 = 1.615$ $s_1 = 0.275$ $n_1 = 4$

(95% confidence interval: $1.178 < \mu_1 < 2.05$)

$\bar{x}_2 = 2.235$ $s_2 = 0.299$ $n_2 = 4$
(95% confidence interval: $1.760 < \mu_2 < 2.710$)

$\bar{x}_3 = 3.053$ $s_3 = 0.439$ $n_3 = 4$
(95% confidence interval: $2.355 < \mu_3 < 3.751$)

Assuming – populations have a normal distribution
– $\varphi_1 \approx \varphi_2 \approx \varphi_3$

$H_0$: $(\mu_1 - \mu_2) = 0$
$H_1$: $\mu_1 \neq \mu_2$

Test statistics:
95% confidence interval $\mu_2 - \mu_1$:

$$(\bar{x}_2 - \bar{x}_1) \pm t_{1/2\alpha} s \sqrt{(1/n_1 + 1/n_2)}$$

where

$$s = \sqrt{\frac{(n_1 - 1)(s_1)^2 + (n_2 - 1)(s_2)^2}{n_1 + n_2 - 2}}$$

*rejection region*: $t_{0.975}$ with $(n_1 + n_2 - 2)$ degrees of freedom.

*Calculation*:

A. group 1 (progressive disease) and 2 (stable disease):

$\bar{x}_2 - \bar{x}_1 = 0.62$

$s = 0.287$

$t_{0.975}$ $(\nu = 6) = 2.45$

$0.123 < \mu_2 - \mu_1 < 1.117$:rejection $H_0$ $(\mu_2 - \mu_1 = 0)$

B. group 2 (stable disease) and 3 (remission)

$\bar{x}_3 - \bar{x}_2 = 0.818$

$s = 0.376$

$t$ $(\nu = 6) = 2.45$

$0.166 < \mu_3 - \mu_2 < 1.469$:rejection $H_0$ $(\mu_3 - \mu_2 = 0)$

Conclusion: assuming a normal distribution of the ratios (of [sCD8]) and roughly the same amount of variation in the different patient groups there is a significant difference ($\alpha = 0.05$) between the means of the ratios of the three different patient groups.

### References

FARRAR, J.J., BENJAMIN, W.R., HILFIKER, M.L., HOWARDS, M., FARRAR, W.L. & FULLER-FARRAR, J. (1982). The biochemistry, biology and role of interleukin-2 in the induction of cytotoxic T cell and antibody-forming B cell responses. *Immunol. Rev.*, **63**, 129–176.

FLEISCHER, B., SCHREZENMEIER, H. & WAGNER, H. (1986). Function of the CD4 and CD8 molecules on human cytotoxic T lymphocytes: regulation of T cell triggering. *J. Immunol.*, **136**, 1625–1628.

FUJIMOTO, J., STEWART, S.J. & LEVY, R. (1984). Immunocytochemical analysis of the released Leu-2 (T8) molecule from human T cells. *J. Exp. Med.*, **160**, 116–124.

GIBLIN, P., LEDBETTER, J.A. & KAVATHAS, P. (1989). A secreted form of the human lymphocyte cell surface molecule CD8 arises from alternative splicing. *Proc. Natl Acad. Sci. USA*, **86**, 990–1002.

GRIFFIN, D.E., WARD, J.W., JAREGUI, E., JOHNSON, R.T. & VAISBERG, A. (1989). Immune activation in measles. *N. Engl. J. Med.*, **320**, 1667–1672.

KNIEP, E.M., STRELOW, I. & LOHMANN-METTHES, M.L. (1992). The monocyte interleukin-2 receptor light chain: production of cell-associated and soluble interleukin-2 receptor by monocytes. *Immunology*, **75**, 299–304.

LISSONI, P., BARNI, S., ROVELLI, F., VIVIANI, S., MAESTRONI, G.J.M., CONTI, A. & TANCINI, G. (1990). The biological significance of soluble Interleukin-2 receptors in solid tumors. *Eur. J. Cancer Clin. Oncol.*, **26**, 33–36.

LOTZE, T.M., CUSTER, M.C., SHARROW, S.O., RUBIN, L.A., NELSON, D.L. & ROSENBERG, S.A. (1987). *In vivo* administration of purified human Interleukin-2 to patients with cancer: development of Interleukin-2 receptor positive cells and circulating soluble Interleukin-2 receptors following Interleukin-2 administration. *Cancer Res.*, **47**, 2188–2195.

PHILIP, T., MERCATELLO, A., NEGRIER, S., PHILIP, I., REBATTU, P., KAEMMERLIN, P., GASPARD, M., TOGNIER, E., COMBARET, V., BIJMANN, J.T., FRANKS, C.R., CHAUVIN, F., MOSKOVTCHENKO, J.F., FAVROT, M. & CLAVEL, M. (1989). Interleukin 2 with or without LAK cells in metastatic renal cell carcinoma: the Lyon first year experience on 20 patients. *Cancer Treatment Rev.*, **16** (Supplement A), 91–104.

ROSENBERG, S.A., LOTZE, M.T., MUUL, L.M., CHANG, A.E., AVIS, F.P., LEITMAN, S., LINEHAN, M., ROBERTSON, C.N., LEE, R.E., RUBIN, J.T., SEIPP, C.A., SIMSON, C.G. & WHITE, D.E. (1987). A progress report on the treatment of 157 patients with advanced cancer using lymphokine-activated killer cells and interleukin-2 or high-dose interleukin-2 alone. *N. Engl. J. Med.*, **316**, 889–897.

RUBIN, L.A., KURMAN, C.C., FRITZ, M.E., BIDDISON, W.E., BOUTIN, B., YARCHOAN, R. & NELSON, D.L. (1985). Soluble Interleukin-2 receptors are released from activated human lymphoid cells *in vitro*. *J. Immunol.*, **135**, 3172–3177.

SAADEH, C., AUZENNE, C., NELSON, D. & ORSON, F. (1986). Sera from the aged contain higher levels of Il-2 receptor compared to young adults. *Fed. Proc.*, **45**, 378.

SLEIJFER, D.TH., JANSSEN, R.A.J., WILLEMSE, P.H.B., MARTENS, A., DE LEIJ, L., DE VRIES, E.G.E. & MULDER, N.H. (1990). Low-dose regimen of interleukin-2 for metastatic renal carcinoma. *The Lancet*, **335**, 1522–1523.

SLEIJFER, D.TH., JANSSEN, R.A.J., BUTER, J., DE VRIES, E.G.E., WILLEMSE, P.H.B. & MULDER, N.H. (1992). Phase II study of subcutaneous interleukin-2 in unselected patients with advanced renal cell cancer on an outpatient basis. *J. Clinical Oncol.*, **10**, 1119–1123.

TOMKINSON, B.E., BROWN, M.C., IP, S.H., CARABIS, S. & SULLIVAN, J.L. (1989). Soluble CD8 during T cell activation. *J. Immunol.*, **142**, 2230–2236.